# Physiological Monitoring Detected Changes During Women's Soccer Anterior Cruciate Ligament Injury

**DOI:** 10.7759/cureus.14838

**Published:** 2021-05-04

**Authors:** John P Detherage, Jon G Divine, Michael A Donaworth, Thomas G Palmer, Joshua A Hagen, Kimberly A Hasselfeld, Marsha Eifert-Mangine, Robert E Mangine, Joseph F Clark, Brian M Grawe

**Affiliations:** 1 Division of Sports Medicine, Department of Orthopaedic Surgery, University of Cincinnati College of Medicine, Cincinnati, USA; 2 Department of Rehabilitation Sciences, University of Cincinnati, Cincinnati, USA; 3 Human Performance Innovation Center, Wright-Patterson Air Force Base, Dayton, USA; 4 Department of Physical Therapy, Mount St. Joseph University, Cincinnati, USA; 5 Department of Athletics, NovaCare Rehabilitation, University of Cincinnati, Cincinnati, USA; 6 Department of Neurology and Rehabilitation Medicine, University of Cincinnati College of Medicine, Cincinnati, USA

**Keywords:** heart rate recovery, autonomic nervous system, anteriort cruciate ligament injury

## Abstract

A growing number of studies utilizing wearable technologies are examining the influence of the autonomic nervous system (ANS) on intense training, recovery, and injury risk. Exercise biometric (EB) data were collected on collegiate, female soccer players during a preseason camp. One player sustained an anterior cruciate ligament (ACL) injury. Baseline anthropometric and EB data were compared to non-injured, position-matched teammates.

All players had similar baseline testing. The injured athlete had a higher body mass index (BMI) and slower vision reaction time (RT). On the day of her injury (DOI), relative percentage heart rate recovery (tHRR) between intense training sets was calculated. Relative percentage tHRR was much lower for the injured athlete, indicating reduced recovery between training sets immediately prior to the injury. Also on DOI, the injured athlete had a lower glomerular filtration rate (GFR).

In addition to BMI and RT differences, the lower relative percentage tHRR and GFR on the DOI observed for the injured athlete may reflect an imbalanced ANS recovery, and potentially to risk factors leading to her ACL injury.

## Introduction

Anterior cruciate ligament (ACL) injury rates continue to rise in National Collegiate Athletic Association (NCAA) women’s soccer players and account for the greatest average number of days lost from participation (159 days) [1,2]. Females are more likely to sustain ACL injuries than males, and their injuries can be attributed to extrinsic risk factors such as playing field surface, or intrinsic factors including hormonal, neuromusculoskeletal fatigue, body composition, and biomechanical differences between men and women [1,3]. Longer reaction time (RT) and generalized joint laxity have also been proposed as injury risk factors [4-7]. Little is known about the quantifiable impact of fatigue on injury occurrence. For this reason, several studies have reviewed coaching and training influences on ACL injury risk with mixed results [1-3].

Wearable technology is commonly utilized by high-level athletes to collect exercise biometrics (EB) or the recording of an athlete’s physiologic response to a given exercise training workload. EB data include, but are not limited to, global positioning systems (GPS) data on distance, speed, acceleration along with heart rate (HR), HR intensity, heart rate variability (HRV), and heart rate recovery (tHRR) [8,9]. The increased ability to collect EB data is having a large impact in the field of sports medicine when it comes to monitoring and forecasting workload and cardiovascular health and assessment of injury risk of athletes on various team sports around the world. However, wearable technology is still limited in terms of injury prediction due to different injury definitions and reported workload metrics, poor study quality, and the lack of accurate and multivariate probabilistic models due to an incomplete understanding of the determinants of an injury [10-12].

This retrospective cohort study aims to examine EB data of an individual ACL tear occurrence and previously reported intrinsic, extrinsic, and fatigue factors resulting in ACL tears in Division I female soccer players [3,8,13].

## Materials and methods

This retrospective cohort study compared an injured NCAA Division I women’s soccer player participating in pre-competitive season training to a group of seven uninjured, female teammates involved in the same training, who served as age and position (forwards) matched controls. All members of the team had several EB values recorded as a part of a larger training study. Baseline anthropometrics including body composition (Bod Pod™); joint hypermobility by Beighton Score; along with central and peripheral vision RTs (DynaVision™) were obtained on each athlete prior to the pre-season training period. Baseline and post-training blood samples for plasma biomarkers associated with overtraining including creatinine (Cr), glomerular filtration rate (GFR), creatine kinase (CK), lactate dehydrogenase (LDH), myoglobin (Myo), cortisol (Cort), estrogen (Est), were obtained at regular five-day intervals throughout training camp, including Day 6. Physiological load (PL) was measured and computed by an algorithm created by the Zephyr Performance System™. The PL increases at a rate proportional to the ratio of the athlete’s current HR in real-time, to their previously determined HR max obtained from a standardized VO2 max test, and a point value is assigned to the percent of the HR max at each time point. The PL continues to accumulate in value throughout the entirety of the athlete’s training session. Distance ran/walked was monitored by combined HR monitoring/GPS device worn by the athletes while training.

## Results

On Day 6 of camp, one of the players, a 20-year-old player training with teammates near the end of a workout made a noncontact foot plant, pivoted, and felt a pop localized to her non-dominant, left knee during a change-in-direction. A Lachman’s exam was positive on the field raising concern of an ACL tear, which was confirmed later the same day with magnetic resonance imaging. Eleven days post-injury, the athlete’s injured ACL was reconstructed using a bone-tendon-bone autograft along with partial excision of her lateral meniscus.

On the day of her injury (DOI), the athlete was on Day 9 of her menstrual cycle (pre-ovulation): days 1-9 have been previously reported as being a lower risk time period for female ACL injury [14]. Her height was 165.1 cm. and weight 70.3 kg, with a body mass index (BMI) of 26.3, the highest of her teammates (outside of the 95th percentile CI). Her baseline reaction test time was outside of the 95th percentile of her teammates. A summary of anthropometric, body composition and baseline RT values are included in Table 1.

**Table 1:** Baseline anthropometric and reaction time testing values for the injured and non-injured athletes. SD: standard deviation; CI: confidence interval **≥ 95% CI for team

Variable	Injured Athlete	Uninjured Athletes (n=7)	
	Value	Mean (SD)	95% CI
Age	20.0	19.3 (1.11)	18.3, 20.3
Height (cm)	165.1	163.3 (5.2)	158.5, 167.6
Weight (kg)	70.3	61.9 (9.3)	53.3, 70.4
Body fat %	26.3**	18.6 (6.29)	12.7, 24.4
Fat weight (kg)	18.5	11.7 (5.0)	5.2, 19.5
Lean weight (kg)	51.8	50.1, (6.4)	45.0, 60.7
Beighton score	3	1.43 (1.27)	0, 3
DynaVision A* (hits/min)	73	70.3 (5.60)	65 ,81
DynaVision Reaction test (secs)	0.49**	0.39 (0.05)	0.32, 0.47

**Figure 1:**
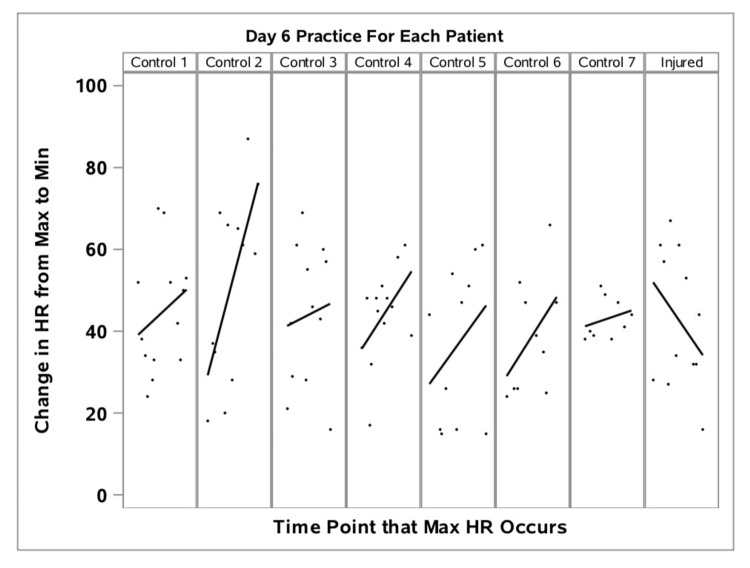
Percent of heart rate maximums (during exercise sets) and minimums (between exercise sets) of injured and control athletes on Day 6 of training camp. HR, heart rate

Based upon EB data (Table 2), the athlete demonstrated a progressively higher relative maximum training HR during exercise sets, with a decreased rate of HR recovery between sets on the DOI than her teammates (Figure 1). Figure 1 represents HR recovery (HRR) during the Day 6 training session for each of the eight athletes: the injured athlete’s relative percentage of HRR during the training session is provided in the far-right column.

**Table 2:** Physiological monitoring EB parameters, peak heart rate, physiological load, and total distance of the injured and non-injured athletes for all workouts during the first 6 days of summer training camp. SD: standard deviation; CI: confidence interval; EB: exercise biometric *≤ 95% CI. **≥ 95% CI.

Monitored variables		Day		Workout of Day	Injured Athlete	Non-injured Athletes (n=7)		Injured Athlete
					Value	Mean (SD)	95% CI	SD from total mean
Peak heart rate (beats per minute)								
	1		1		189	190.7 (2.98)	183.6, 197.8	-0.57
	1		2		190	195.1 (5.6)	181.9, 208.3	-0.91
	2		1		182	182.3 (5.44)	169.4, 195.2	-0.06
	2		2		178	182.6 4.12)	172.7, 192.3	-1.12
	3		1		177	188.9 (9.44)	166.6, 211.2	-1.26
	3		2		186	192.3 (9.77)	169.2, 215.4	-0.64
	5		1		197	195.9 (5.6)	182,7, 209.1	0.20
	6		1		204**	179.4 (5.9)	165.4, 193.4	4.17**
Physiological load (unitless)								
	1		1		457	657 (111)	395, 918	-1.81
	1		2		406*	646 (74)	470, 822	-3.22*
	2		1		347	626 (140)	296, 956	-2.00
	2		2		149	320 (80)	132, 508	-2.15
	3		1		191	408 (93)	187, 628	-2.32
	3		2		247*	379 (52)	257, 501	-2.56*
	5		1		478	697 (163)	312, 1083	-1.34
	6		1		282	343 (96)	116, 570	-0.64
Total distance (miles)								
	1		1		3.16	3.12 (0.39)	2.20, 4.04	0.10
	1		2		5.18	5.19 (1.46)	1.74, 8.64	-0.01
	2		1		4.55	4.45 (0.60)	3.03, 5.87	0.17
	2		2		2.80	2.93 (0.39)	2.01, 3.85	-0.33
	3		1		2.72	2.97 (0.26)	2.36, 3.58	-0.96
	3		2		2.95	2.98 (0.25)	2.39, 3.57	-0.12
	5		1		5.98	5.96 (0.37)	5.08, 6.84	0.05
	6		1		2.71	2.27 (0.23)	1.73, 2.81	1.91

**Figure 2:**
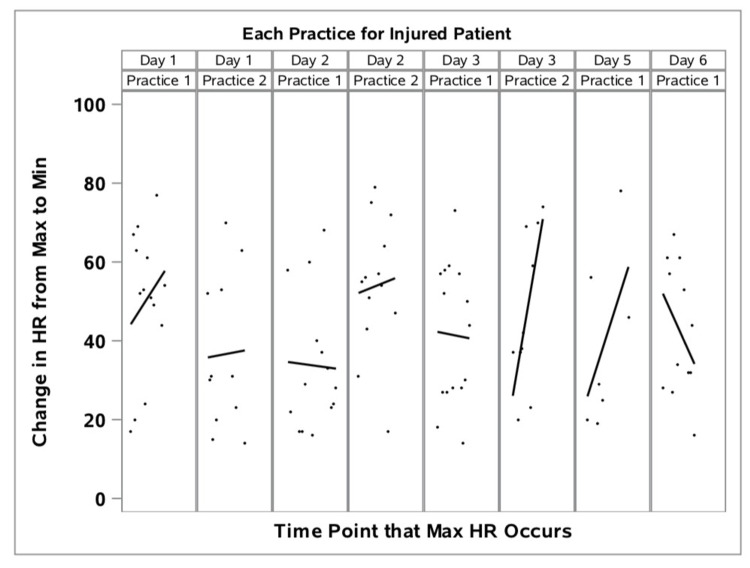
Percent of heart rate maximums (during exercise sets) and minimums (between exercise sets) of injured athlete for training sessions on Days 1-6 of training camp.

Using this calculation, a higher number represents a faster rate of HR recovery. On the DOI, the relative percentage change in HR from the maximum during intense exercise “sets” to a minimum (tHRR) between exercise “sets” continued to decrease, while uninjured athletes were able to return to tHRR levels between sets as indicated by a higher value (Figure 1). Not only did her recovery rate decrease, her maximum HR during sets and trough recovery HR at rest between sets both continued to increase during the workout. This was a very different HR response for the injured athlete on the DOI from the previous five training days in which the injured athlete’s tHRR was similar to the HR recovery patterns of the uninjured teammates which reflected a more rapid rate of HR recovery and lower relative max and trough HR (Figure 2).

Despite having had more cardiovascular fatigue on the DOI, the athlete had no differences in muscle breakdown byproducts on Day 6 than the control-matched teammates; with the exception of having the greatest change in GFR from baseline. Table 3 includes data for plasma biomarkers obtained on training camp Day 0 and Day 6, the day of the injured athlete’s ACL injury. It is important to note that on the night before DOI (Day 5), there was an intense scrimmage in which all eight athletes had participated with similar intensity and duration. Despite following similar post-match recovery practices (lower intensity exercise, fluid, and dietary replacement) and pre-training warm-up, two specific biomarker differences stood out for the injured athlete: greater reduction in GFR and a limited rate of HR recovery between training sets on the DOI.

**Table 3:** Baseline and post-training plasma biomarkers. ^#^19.7% in Glomerular filtration rate

Baseline values	Injured athlete	Uninjured teammates median and 25-75%ile range	Absolute range 0-100%ile	Normal ranges
Creatinine (mg/dL)	0.95	0.83 (0.77, 0.97)	0.76, 1.03	0.6-1.3
Glomerular filtration rate (mL/min/1.73m^2^)	91	88 (74, 97)	83, 117	>90
Creatine kinase (U/L)	154	126 (114, 155)	75, 212	30-223
Lactate dehydrogenase (U/L)	195	173 (149, 193)	136, 197	110-270
Myoglobin (ng/mL)	22.7	14.0 (11.7, 16.1)	8.6, 23.7	14.3-65.8
Day 6 - values				
Creatinine (mg/dL)	1.15	1.04 (0.9, 1.29)	0.85, 1.32	0.6 -1.3
Glomerular filtration rate (mL/min/1.73m^2^)	73^#^	83 (64, 99)	63, 103	>90
Creatine kinase (U/L)	460	333 (460, 841)	308, 2262	30-223
Lactate dehydrogenase (U/L)	244	225 (212, 265)	206, 477	110-270
Myoglobin (ng/mL)	60.1	119 (83, 262)	10, 323	14.3-65.8

## Discussion

Current practices allow for EB data to be monitored and recorded in real-time with the capability to analyze trends in real-time during workouts or over longer time periods during training cycles. In this manner, overtraining is often identified and methods that allow athletes to maximize recovery can be implemented. This is a unique retrospective cohort study comparing the pre-injury training data between a collegiate female soccer player with a non-contact, ACL injury and her position-matched, uninjured teammates. Based upon position-matched comparisons, the injured athlete’s higher baseline BMI and RT along with a greater reduction in GFR and lower tHRR during the workout on the day of injury, differentiated her from her teammates while cumulative plasma biomarkers and baseline joint hypermobility did not.

Baseline differences in body composition and RTs were observed between the injured athlete and her teammates. Studies of age-, and activity-matched athletes indicate that athletes with a higher BMI and relative percentage of body fat are at greater risk of non-contact ACL injury [7,15]. In addition to body composition differences, the injured athlete had a significantly slower baseline RT. There is no reason to believe that her RT improved over the 6 days of training: especially on the DOI when she appeared to have had an incomplete recovery from the prior scrimmage. Her baseline RT was nearly 0.5 sec longer than a visual stimulus which is outside the 95% CI for her teammates. Slower RT is consistent with previous reports that it was associated with a higher risk of non-contact ACL injuries [3,5,13]. There is currently moderate evidence for the relationships between the training load applied to an athlete and the occurrence of injury, and more specifically soft-tissue injury, especially in periods of acute spikes of loads, or the preseason when athletes workloads are generally higher [16,17]. Due to this, we believe the intensity of the preseason scrimmage the night before the injury played a significant role in her injury.

Reviewing individual EB data over the days leading up to the DOI, the injured athlete’s response to daily workout loads were similar to, or slightly lower than her teammates - suggesting the athlete was equally, or slightly more fit than her teammates. Even on the DOI (Day 6), her physiologic load (a unitless, proprietary measure of exercise load developed by Zephyr Performance Systems™) and GPS distance were similar to her teammates. However, her training HR response was much higher. She achieved her highest absolute HR of the entire six-day training period during the workout on Day 6 - just before her ACL injury occurred. Her maximum HR of 204/bpm exceeded the 95% CI for her teammates for the same workout. Of greater importance, her tHRR - a measure of relative recovery within a workout - was much higher than her teammates (Figure 1), indicating an incomplete HR recovery capability between intense sets within the workout.

Also on the DOI, the injured athlete’s GFR had decreased almost 20% from baseline. This reduction in GFR (was second highest of the cohort) did not appear to reflect changes associated with training, or acute exercise-induced rhabdomyolysis, as both the injured athlete and her teammates had very similar changes in post-training CPK and myoglobin. The significance of this decrease GFR is unclear but may reflect increased stress on the kidneys due to lower blood volume as well as an increased sympathetic nervous system (SNS) influence on renal circulation, well known for lowering GFR [18].

There is a significant influence of the autonomic nervous system (ANS) on multiple biosystems: most specifically the cardiovascular, renal and musculoskeletal systems of the athlete during training. When an athlete is training at or near max HR during intense exercise, the SNS has the predominant effect on HR and cardiac function. In the immediate period following intense exercise, there is a rapid decrease in HR that is due to cardiac parasympathetic (PNS) reactivation. Following an intense set of exercises, the rate of return to ANS homeostasis is directly dependent upon the relative intensity of the set [8]. When an athlete in training has not returned back to a balanced ANS effect on HR, it is because of a predominant SNS influence on HR (and other biosystems). With an SNS predominance, HR response to a given exercise load as well as HR recovery during and after exercise training is higher [9]. In addition to the SNS influence on the heart following intense exercise, there is a well-known local efferent SNS influence on the regulation of blood flow to the kidneys resulting in a reduction in GFR, felt to be a protective mechanism done to maintain circulating blood (plasma) volume during periods of intense exercise [18]. Finally, less is known about the direct SNS influence on the musculoskeletal system than upon the cardiac and renal systems; however, the distribution of SNS efferent-nerve content within a ligament has been histologically measured to be 40% of the total innervation and are predominantly involved in blood flow regulation to the ligament, thus indicating the SNS has a large influence upon the musculoskeletal system as well [19,20]. Given this potential degree of SNS effect, it is interesting to speculate that an SNS-driven change in ligament blood flow may play a direct role in the structural integrity of a ligament, specifically the ACL in this case.

Having demonstrated several biometric markers indicating an SNS predominance or an incomplete recovery of ANS equilibrium as her matched, uninjured teammates, perhaps the athlete’s risk of injury was an individual influence of both baseline risk and added risk as a result of incomplete recovery from having participated in an intense scrimmage on the evening prior to the DOI.

## Conclusions

We speculate that this incomplete return to ANS equilibrium was a reflection of relative physical fatigue, which represented a unique risk factor for injury. The aim of this study was to review how the EB resulting from a pre-season training volume can potentially contribute to injury risk. We conclude that observed differences between the injured athlete and her teammates included baseline body fat%, RT, and physical fatigue - objectively based upon higher tHRR on the DOI - and were all potential risk factors leading to her ACL injury. We also conclude that PMB and joint hypermobility were not risk factors for this athlete’s injury. As a result of having an intense scrimmage on the evening prior to the injury, and when compared to her teammates we conclude that the athlete had not completely recovered manifesting as a higher tHRR and reduced GFR. Speculation can be made that the same SNS influences on exercising HR, tHRR, and GFR may have altering effects on ligamentous stability resulting in an increased risk of injury at the local level, in addition to other potential biomechanical stresses being placed upon the knee. Limitations of our study include only being able to study a single injury occurrence and the relatively small sample size of the study.

Further research is needed on the potential effects of EB data and injury prevention. Monitoring the cause and effect relationship between real-time tHRR values and physical training workloads may potentially expand our understanding of the relationships between injury risk, EB data, and recovery methods. Such insight may assist in improving performance and reduce injury risks.
